# Self in NARS, an AGI System

**DOI:** 10.3389/frobt.2018.00020

**Published:** 2018-03-12

**Authors:** Pei Wang, Xiang Li, Patrick Hammer

**Affiliations:** ^1^Department of Computer and Information Sciences, Temple University, Philadelphia, PA, United States

**Keywords:** general intelligence, non-axiomatic logic, self-awareness, self-control, self-organization, consciousness

## Abstract

This article describes and discusses the self-related mechanisms of a general-purpose intelligent system, NARS. This system is designed to be adaptive and to work with insufficient knowledge and resources. The system’s various cognitive functions are uniformly carried out by a central reasoning-learning process following a “non-axiomatic” logic. This logic captures the regularities of human empirical reasoning, where all beliefs are revisable according to evidence, and the meaning of concepts are grounded in the system’s experience. NARS perceives its internal environment basically in the same way as how it perceives its external environment although the sensors involved are completely different. Consequently, its self-knowledge is mostly acquired and constructive, while being incomplete and subjective. Similarly, self-control in NARS is realized using mental operations, which supplement and adjust the automatic inference control routine. It is argued that a general-purpose intelligent system needs the notion of a “self,” and the related knowledge and functions are developed gradually according to the system’s experience. Such a mechanism has been implemented in NARS in a preliminary form.

## 1 Introduction

Phenomena and functions like “self-awareness,” “self-control,” “self-reference,” and “self-consciousness” are closely related to human intelligence, cognition, and thinking, and the related topics have been discussed in various fields (Hofstadter, 1979; Blackmore, 2004).

In the study of artificial intelligence (AI), although these issues have been addressed by the pioneers (Simon, 1962; Minsky, 1985; McCarthy, 1995), they nevertheless have been rarely considered in the technical works, as shown by the lack of coverage of these topics in the common textbooks (Luger, 2008; Russell and Norvig, 2010; Poole and Mackworth, 2017). The difficulty of realizing these functions in a machine is both technical and theoretical, as there is no widely accepted theory about them, and even their definitions are highly controversial.

On the contrary, researchers in the emerging field of artificial general intelligence (AGI) widely consider these functions as necessary for general intelligence and have proposed various ways to cover hem in AGI systems (Schmidhuber, 2007; Baars and Franklin, 2009; Bach, 2009; Shapiro and Bona, 2010; Chella and Manzotti, 2012; Thórisson, 2012; Goertzel, 2014; Rosenbloom et al., 2016). As these approaches are based on very different considerations and typically tangled with the other functions in the system, it is hard to compare them to say which one is the best.

The focus of this article is the relevant aspects of NARS (non-axiomatic reasoning system), a formal model of general intelligence, which has been mostly implemented and is under testing and tuning. In the following, the conceptual design of NARS is introduced first, then the parts mostly relevant to “self” are described in more detail. Finally, the major design decisions are compared with the related works.

## 2 NARS Overview

NARS (non-axiomatic reasoning system) is an AGI-designed framework of a reasoning system. The project has been described in many publications, including two books (Wang, 2006, 2013), so it is only briefly summarized here.

### 2.1 Theoretical and Strategic Assumptions

The working definition of “intelligence” in NARS is different from that in mainstream AI, where “Intelligence” is usually taken as an ability to solve problems that are only solvable by the human brain. A computer agent can obtain this ability by developing domain-specific solutions. Instead, NARS is designed according to the belief that “Intelligence” is *the ability for a system to adapt to its environment and to work with insufficient knowledge and resources*. It requires the system to have the capacities of accepting unanticipated problems and events, making real-time responses, working with finite resources, and learning from its experience in an application domain.

The behaviors of NARS are based on past experiences and generated by interacting with the environment in real time; therefore, the solutions provided by the system to the problems are usually not the optimum solutions but the best solution that the system can find at the moment. The system could always do better with more resources and knowledge, especially in a relatively stable environment. Compared to the other theories of rationality, the most significant feature of this “relative rationality” is the *Assumption of Insufficient Knowledge and Resources*, hereafter **AIKR**. Concretely, the following three features are demanded by AIKR, with respect to the problems to be solved by the system:

- **Finite:** The system is able to work with constant information-processing capacity, in terms of processor speed, storage space, etc.
- **Real time:** The system is able to deal with problems that show up at any moment and the utility of their solutions may decrease over time.
- **Open:** The system is able to accept input data and problems of any content, as long as they are expressed in a format recognizable by the system.

Due to the time and resources restriction, and also the uncertainty about the coming problems, NARS usually cannot consider all possibilities when facing a problem, but will only consider some important and relevant possibilities, judged according to the system’s experience.

According to AIKR, NARS does not treat the storage space of itself as infinite. The mechanism of forgetting is a special feature of NARS to deal with limited storage space. Some beliefs or tasks will be removed from the storage of NARS when their *priority* (to be introduced later) is below a threshold.

AIKR is a fundamental assumption abstracted from the study of human problem solving in the real world. Humans obtain knowledge by learning and summarizing past experience. When humans deal with problems that they do not know how to solve at the moment, they will attempt to solve them with the help of relevant knowledge. *This ability is exactly what we consider intelligence, which is characterized not by what problems it can solve, but the restriction under which the problems are solved*.

The research goal of NARS is to design and build a computer system that can adapt to its environment and solve problems under AIKR. This is different from the objectives of the mainstream AI projects, which are specific problem-solving abilities. The aim of NARS is to build a system with a given learning ability (at the meta-level) that allows the system to acquire various problem-solving skills from its experience.

Although being a reasoning system is neither a necessary nor sufficient condition for being intelligent, a reasoning system can provide a suitable framework for the study of intelligence, as it forces the system to be general purpose, instead of being domain specific. Reasoning is at a more abstract level than other low-level cognitive activities, and it is obviously a critical cognitive skill that qualitatively distinguishes human beings from other animals.

Many cognitive processes such as planning, learning, decision-making, etc., can be formulated as types of reasoning; therefore, an intelligent system designed in the framework of a reasoning system can be extended to cover them easily. As a reasoning system follows a logic, each step of processing must be justifiable independently. As a result, inference steps can be linked at run time in novel orders to handle novel problems. This is a major reason why NARS is designed as a reasoning system.

### 2.2 Knowledge Representation

As a reasoning system, NARS uses a formal language called “Narsese” for knowledge representation, which is defined by a formal grammar given in the study by Wang (2013). To fully specify and explain this language is beyond the scope of this article, so in the following, only the directly relevant part is introduced informally and described briefly.

The logic used in NARS belongs to a tradition of logic called “term logic,” where the smallest component of the representation language is a “term,” and the simplest statement has a “subject-copula-predicate” format, where the subject and the predicate are both terms.

The basic form of statement in Narsese is *inheritance statement*, which has a format “*S* → *P*,” where S is the subject term, and P is the predicate term, the “→” is the *inheritance* copula, which is defined as a reflexive and transitive relation from one term to another term. The intuitive meaning of “*S* → *P*” is “S is a special case of P” and “P is a general case of S.” For example, statement “*robin* → *bird*” intuitively means “Robin is a type of bird.”

We define the *extension* of a given term T to contain all of its known special cases and its *intension* to contain all of its known general cases. Therefore, “*S*→*P*” is equivalent to “S is included in the extension of P,” and “P is included in the intension of S.”

The simplest, or “atomic,” form of a term is a *word*, that is, a string of characters from a finite alphabet. In this article, typical terms are common English nouns like *bird* an *animal*, or mixed by English letters, digits 0–9, and a few special signs, such as hyphen(“-”) and underscore (“_”), but the system can also use other alphabets or use terms that are meaningless to human beings, such as “drib” and “aminal.”

Beside atomic terms, Narsese also includes *compound terms* of various types. A compound term (*con, C*_1_, *C*_2_, …, *C_n_*) is formed by a term connector, *con*, and one or more component terms (*C*_1_, *C*_2_, …, *C_n_*). The term connector is a logical constant with predefined meaning in the system. Major types of compound terms in Narsese include the following:

- **Sets:** Term {*Tom, Jerry*} is an *extensional set* specified by enumerating its instances; term [*small, yellow*] is an *intensional set* specified by enumerating its properties.
- **Intersections and differences:** Term (*bird* ∩ *swimmer*) represents “birds that can swim”; term (*bird* – *swimmer*) represents “birds that cannot swim.”
- **Products and images:** The relation “John is the uncle of Zack” is represented as “({*John*} × {*Zack*}) → *uncle-of*,” “{*John*}→ (*uncle-of* / ◊ {*Zack*}),” and “{*Zack*}→ (*uncle-of* / {*John*} ◊),” equivalently.^1^ Here, ◊ is a placeholder, which indicates the position in the *uncle-of* relation the subject term belongs to.
- **Statement:** “John knows soccer balls are round” can be represented as a *higher-order statement* “{*John*}→ (*know* / ◊ {*soccer-ball* → [*round*]}),” where the statement “*soccer-ball *→* [round]*” is used as a term.
- **Compound statements:** Statements can be combined using term connectors for disjunction(“∨”), conjunction(“∧”), and negation(“¬”), which are intuitively similar to those in propositional logic, but not defined using truth-tables.^2^

Several term connectors can be extended to take more than two component terms, and the connector is often written before the components rather between them, such as (× {*John*}{*Zack*}).

Beside the *inheritance* copula (“→”, “is a type of”), Narsese also has three other basic copulas: *similarity* (“↔”, “is similar to”), *implication* (“⇒”, “if-then”), and *equivalence* (“⇔”, “if-and-only-if”), and the last two are used between statements.

In NARS, an *event* is a statement with temporal attributes. Based on their occurrence order, two events *E*_1_ and *E*_2_ may have one of the following basic temporal relations:

- *E*_1_ happens before *E*_2_
- *E*_1_ happens after *E*_2_
- *E*_1_ happens when *E*_2_ happen

More complicated temporal relations can be expressed by taking about the subevents of the events.

Temporal statements are formed by combining the above basic temporal relations with the logical relations indicated by the term connectors and copulas. For example, implication statement “*E*_1_ ⇒ *E*_2_” has three temporal versions, corresponding to the above three temporal orders, respectively^3^:

- *E*_1_ /⇒ *E*_2_
- *E*_1_ \⇒ *E*_2_
- *E*_1_ |⇒ *E*_2_

All the previous statements can be seen as Narsese describing things or events from a third-person view. Narsese can also describe the actions of the system *itself* with a special kind of event called *operation*. An operation is an event directly realizable by the system itself *via* executing the associated code or command.

Formally, an operation is an application of an operator on a list of arguments, written as *op*(*a*_1_, …, *a*_n_) where *op* is the operator, and *a*_1_, …, *a*_n_ is a list of arguments. Such an operation is interpreted logically as statement“(× {*SELF*} {*a*_1_} … {*a_n_*}) → *op*,” where *SELF* is a special term indicating the system itself, and *op* is an operator that has a procedural interpretation. For instance, if we want to describe an event “The system is holding key_001,” the statement can be expressed as “(× {*SELF*} {*key*_001})→ *hold*.”

Overall, there are three types of sentences defined in Narsese:

- A **judgment** is a statement with a truth value and represents a piece of new knowledge that system needs to learn or consider. For example, “*robin* → *bird* ⟨*f, c*⟩,” where the truth value ⟨*f, c*⟩ will be introduced in the next section.
- A **question:** is a statement without a truth value, and represents a question to be answered according to the system’s beliefs. For example, if the system has a belief “*robin* → *bird*” (with a truth value), it can be used to answer question “*robin* → *bird*?” by reporting the truth value, as well as to answer the question “*robin* → ?” by reporting the truth value together with the term *bird*, as it is in the intension of *robin*. Similarly, the same belief can also be used to answer question “? → *bird*” by reporting the truth value together with the term *robin*.
- A **goal** is statement without a truth value, and represents a statement to be realized by executing some operations, according to the system’s beliefs. For example, “(× {*SELF*} {*door*_001}) → *open*!” means the system has the goal to open the *door*_001 or to make sure that *door*_001 is opened. Each statement of goal always associates with a “desire-value,” indicating the extent to which the system hopes for a situation where the statement is true.

The *experience* of NARS consists of a stream of input sentences of the above types.

### 2.3 Experience-Grounded Semantics

When studying a language, semantics relates the items in the language to the environment in which the language is used. It answers questions like “What is the meaning of this term?,” or “What is the truth value of that statement?”

Since NARS is designed under AIKR, the truth value of a statement measures its extent of evidential support, rather than that of agreement with a corresponding fact. NARS does not determine the truthfulness of its knowledge with respect to a static and completely described environment. Since the environment changes over time, there is no guarantee that the past is always identical to the future. Hence, in NARS, the truth of each statement and the meaning of each term are grounded on nothing but the system’s experience. The formal definition of this semantics and discussions of its implications can be found in the studies by Wang (2005, 2013) and are only briefly summarized in the following.

As mentioned previously, in Narsese, “*robin* → *bird*” states that “Robin is a type of bird,” and it is equivalent to saying that the extension of *robin* is included in the extension of *bird*, as well as the intension of *bird* is included in the intension of *robin*. Therefore, if a term is in the extension (or intension) of both *robin* and *bird*, then its existence supports the statement or provides positive evidence. On the contrary, if a term is in the extension of *robin* but not the extension of *bird*, or is in the intension of *bird* but not the intension of *robin*, it provides negative evidence for the statement.

For a given statement, we use *w^+^, w^–^*, and *w* to represent the amount of positive, negative, and total evidence, respectively. Based on them, a two-dimensional truth value is defined as a pair of real numbers ⟨*f, c*⟩ for the measurements. Here, *f* is called the *frequency* of the statement and is defined as the proportion of positive evidence among total evidence, that is, *f* = *w*^+^/*w*. The value *c* is called the *confidence* of the statement and is defined as the proportion of current evidence among total amount of evidence at a moment in the future after new evidence of a certain amount is collected, that is, *c* = *w*/(*w* + *k*), where *k* ≥ 1. This constant *k* is a “personality parameter” and is explained further in the study by Wang (2013). The value of *k* can be seen as a unit of evidence that decides how fast the *c* value increases as new evidence comes, and in the following, we use the default *k* = 1 to simplify the discussion. Roughly speaking, *frequency* represents the uncertainty of the statement, and *confidence* represents the uncertainty of the frequency (Wang, 2001). Defined in this way, *truth value* in NARS is “experience-grounded.”

Similarly, the *meaning* of a term is defined as its extension and intension, so it is determined by how it is related to other terms in the system’s experience. As the experience of a system grows over time, the truth value of statements and the meaning of terms in the system change accordingly. This *experience-grounded semantics* (EGS) is fundamentally different from the traditional *model-theoretic semantics*, since it defines *truth value* and *meaning* according to a (dynamic and system-specific) experience, rather than a (static and system-independent) model. In the simplest implementation of NARS, its *experience* is a stream of Narsese sentences, which will be summarized to become the system’s *beliefs*, which is also called the system’s *knowledge*. This semantics is formally defined and fully discussed in the study by Wang (2005, 2006).

### 2.4 Inference Rules

The logic followed by NARS is NAL (non-axiomatic logic), and its inference rules use Narsese sentences as premises and conclusions. A recent version of NAL is formalized and justified in the study by Wang (2013). What is described in the following is only a small part of NAL that is directly related to the current topic.

NAL uses formal inference rules to recursively derive new knowledge from existing knowledge, which consists of statements with truth values, indicating the experienced relations between terms and the strength of these relations. Each inference rule has a truth value function that calculates the truth value of the conclusion according to the evidence provided by the premises.

In terms of the type of reasoning, inference rules of NARS are divided into three categories:

- **Local rules:** These rules do not derive new statements. Instead, the conclusion comes out from a revision or selection of the premises.
- **Forward rules:** New judgments are produced from a given judgment and a relevant belief.
- **Backward rules:** New questions (or goals) are produced from a given question (or goal) and a relevant belief.

In the following, these three groups of rules are introduced in that order.

Under AIKR, NARS may have inconsistent beliefs, that is, the same statement may obtain different truth values according to different evidential bases. When the system locates such an inconsistency, it either uses the *revision* rule (if the evidence bases are disjoint) or the *choice* rule (if the evidence bases are not disjoint). The revision rule accepts two judgments about the same statement as premises and generates a new judgment for the statement, with a truth value obtained by pooling the evidence of the premises. Consequently, the frequency of the conclusion is a weighted sum of those premises, and the confidence is higher than those of the premises. The choice rule simply choose the premise that has more positive evidence and less negative evidence, while preferring simpler candidates.^4^

As a term logic, typical inference rules in NAL are *syllogistic*, and each rule takes two premises (with one common term) to derive a conclusion (between the other two terms). The NAL rules of this type include *deduction, induction*, and *abduction*, similar to how the three are specified by Peirce (1931), although the truth value of every statement is extended from {0, 1} to [0,1] × (0,1). These three inference rules are the most basic forward rules of NAL, where *M, P*, and *S* represent arbitrary terms:

**Table:** 

Deduction	Induction	Abduction
M → P ⟨*f*_1_, *c*_1_⟩	M → P ⟨*f*_1_, *c*_1_⟩	P → M ⟨*f*_1_, *c*_1_⟩
S → M ⟨*f*_2_, *c*_2_⟩	M → S ⟨*f*_2_, *c*_2_⟩	S → M ⟨*f*_2_, *c*_2_⟩

S → P ⟨*f, c*⟩	S → P ⟨*f, c*⟩	S → P ⟨*f, c*⟩

Different forward inference rules have different truth value functions that calculate ⟨*f, c*⟩ from ⟨*f*_1_, *c*_1_⟩ and ⟨*f*_2_, *c*_2_⟩. These functions are established in the study by Wang (2013), and here, we do not describe the actual functions, but merely divide the inference rules into two groups, according to the maximum *confidence* value of the conclusions:

- **Strong inference:** The upper bound of confidence is 1. Among the rules introduced so far, only the *deduction* rule belongs to this group.
- **Weak inference:** The upper bound of the confidence is 1/(1 + *k*) ≤ 1/2. The *abduction* and *induction* rules belong to this group.

The *weak inference* rules in NARS usually carry out *learning*, where each piece of evidence generates a weak conclusion, and strong conclusions are accumulated by the *revision* rule from many weak conclusions. This is why “learning” and “reasoning” are basically the same process in NARS (Wang and Li, 2016).

NAL has other syllogistic rules and also has *compositional* rules to build compound terms to capture the observed patterns in experience. For example, from “*swan* → *bird* ⟨*f*_1_, *c*_1_⟩” and “*swan* → *swimmer* ⟨*f*_2_, *c*_2_⟩,” a rule can produce “*swan* → (∩, *bird, swimmer*) ⟨*f, c* ⟩.”

The inference rules of NAL can be used in both *forward inference* (from existing beliefs to derived beliefs) and *backward inference* (from existing beliefs and questions/goals to derived questions/goals). For each forward inference rule that from two judgments *J*_1_ and *J*_2_ to derive a conclusion *J*, a backward inference rule can be established that takes *J*_1_ and a question on *J* as input and derives a question on *J*_2_, because an answer for the derived question can be used together with *J*_1_ to provide an answer to the original question. For example, if the question is “*robin* → *animal*?,” and there is a related belief “*robin* → *bird* ⟨*f, c*⟩,” then a derived question “*bird* → *animal*?” can be generated. The backward inference on goals is similar.

### 2.5 Inference Control

Equipped with the inference rules of NAL, NARS can carry out the following types of inference tasks:

- To absorb new experience into the system’s beliefs, as well as to spontaneously derive some of their implications.
- To answer the input questions and the derived questions according to the system’s beliefs.
- To achieve the input goals and the derived goals by executing the related operations according to the system’s beliefs.

Under AIKR, new tasks can enter the system at any time, each with its own time requirement, and its content can be any Narsese sentence. Working in such a situation, usually NARS cannot perfectly accomplish all tasks in time, but has to allocate its limited time and space resources among them and to dynamically adjust the allocation according to the change of context, the feedback to its actions, and other relevant factors.

In the memory of NARS, beliefs and tasks are organized into *concepts*, according to the terms appearing in them. Roughly speaking, for a term *T*, concept *C_T_* refers to all beliefs and tasks containing *T*. For example, the beliefs on “*robin* → *bird*” are referred to within concepts *C_robin_* and *C_bird_*, as well as other relevant concepts. A “concept” in NARS is a unit of both storage and processing and models the concepts found in human thinking.

To indicate the relative importance of concepts, tasks, and beliefs to the system, *priority* distributions are maintained among them. The priority of an item (concept, task, or belief) summarizes the attributes to be considered in resource allocation, including its intrinsic quality, usefulness in history, relevance to the current context, etc. Consequently, items with higher priority values will get more resources.

*Bag* is a data structure specially designed for resource allocation in NARS. A certain type of data items is contained in a bag with a constant capacity, with a priority distribution among the items maintained. There are three basic operations defined in a bag:

- *put(item)*: put an item into the bag, and if the bag is full, remove an item with the lowest priority
- *get(key)*: take an item from the bag with a given key that uniquely identifies the item
- *select()*: select an item from the bag, and the probability for each item to be selected is positively correlated with its priority value

NARS works by repeating an inference cycle consisting of the following major steps:

1. Select a concept within the memory
2. Select a task referred by the concept
3. Select a belief referred by the concept
4. Derive new tasks from the selected task and belief by the applicable inference rules
5. Adjust the priority of the selected belief, task, and concept according to the context and feedback
6. Selectively put the new tasks into the corresponding concepts and report some of them to the user

All selections in the above steps are probabilistic, and the probability for an item to be selected is positively correlated to its priority value. Consequently, the tasks will be processed in a time-sharing manner, with different speeds. For a specific task, its processing does not follow a predetermined algorithm, but it is the result of many inference steps, whose combination is formed at run time, so is usually neither predictable nor repeatable accurately, as both the external environment and the internal state of the system change in a non-circular manner.

## 3 “Self” in NARS

In this section, we focus on the aspects of NARS that are directly relevant to self-awareness and self-control.

### 3.1 Self in Various Forms

“Self” takes multiple forms in NARS. Some of the relevant properties are addressed by different mechanisms built into the system, and some others are shown in the system’s learning process, including the following:

- **Higher-order statements**: As described previously, the higher-order statements in Narsese cover “statement about statement,” “knowledge about operations,” etc., which are often taken as functions of “metacognition” (Cox, 2005). Since such knowledge is typically about individual statements or operations and not about the system as a whole, and they are not the focus of this article. This type of knowledge usually is processed using inference rules analogical to these used on the statement level. For more details, see the studies by Wang (2006, 2013).
- **Intrinsic mechanisms:** As a part of the inference control process, NARS constantly compares the certainty of beliefs and dynamically allocates its resources among competing tasks. Even though the relevant mechanisms are indeed at a meta-level with respect to beliefs and tasks, they are implicitly embedded in the code, so not generally accessible to the system’s deliberation nor can they be modified by the system itself. Therefore, they describe a constant aspect of the system itself that is not reflected in the object-level beliefs of the system.
- **Experience-grounded semantics:** As mentioned previously, the system’s beliefs and concepts are built from the viewpoint of the system itself rather than as an objective model of the world. In this sense, all beliefs in NARS are subjective, and all concepts have idiosyncratic meanings to various extents. Consequently, the system’s behaviors can be explained and predicted only when the unique experience of the system itself is taken into consideration.

Although the above mechanisms are all related to the system itself in a broad sense, they nevertheless can be described without explicitly using the notion of “self.” In the following, the discussion will focus on “self” in a narrow sense, where a reference to the system as a whole becomes necessary.

### 3.2 The “Self”-Concept

NARS’ beliefs about itself start at its built-in operations. As mentioned above, operation *op*(*a*_1_, …, *a_n_*) corresponds to a relation that the system can establish between itself and the arguments, so it is equivalent to statement “(×{*SELF*} {*a*_1_}…{*a_n_*}) → *op*” (where the subject term is a *product* term written in the prefix format), since it specifies a relation among the arguments plus the system identified by the special term *SELF*.

Similar to the case of logic programming (Kowalski, 1979), here the idea is to uniformly represent declarative knowledge and procedural knowledge. So in NARS, knowledge about the system itself is unified with knowledge about others. For instance, the operation “open this door” is represented as “(×{*SELF*}{*door*_1}) → *open*,” so the inheritance copula encodes that the relation between {*SELF*} and {*door*_1} is a special case of opening. On the other hand, “John opened this door” is represented as “(×{*John*}{*door*_1}) → *open*” (tense omitted to simplify the discussion). In this way, imitation can be carried out by analogical inference.

According to experience-grounded semantics (EGS), in NARS, the meaning of a concept is gradually acquired from the system’s experience. However, EGS does not exclude the existence of innate concepts, beliefs, and tasks. In the above example, *SELF* is such a concept, with built-in operations that can be directly executed from the very beginning of the system’s life. Such operations depend on the hardware/software of the host system, so are not specified as parts of NARS, except that they must obey the format requirements of Narsese. According to EGS, in the initial state of NARS, the meaning of a built-in operation is procedurally expressed in the corresponding routine, while the meaning of *SELF* consists of these operations. To the system, “*I* am whatever I can do and feel,” since in NARS *sensation* (converting signals into terms) and *perception* (organizing terms into compounds) are also carried out by operations.

As the system begins to have experience, the meaning of every concept will be more or less adjusted as it is experienced, directly or indirectly. For a built-in operation, the system will gradually learn its preconditions and consequences, so as to associate it with the goals it can achieve and the context where it can be used. It is like we learn how to raise our hand first and then know it as a way to get the teacher’s attention. The *SELF*-concept will be enriched in this way, as well as through its relations with other concepts representing objects and other systems in the outside environment.

Therefore, self starts from “what I can do and feel” to include “what I am composed of,” “how I look like,” “what my position is in the society,” etc. The notion “self” does not have a constant meaning determined by a denotation or definition. Instead, the system gradually learns who it is, and its self-image does not necessarily converge to a “true self.” Since the change of meaning of a concept is done *via* the additions, deletions, and revisions of its relations with other concepts, the system’s identity (determined by all the relations) is relatively stable in a short period, although in its whole life the system may change greatly, even to the extent of unrecognizable when compared to a previous image of itself. Under AIKR, the system is open to all kinds of experience, so in the design of NARS, there is no restriction on the extent of these changes.

When NARS is used to serve a practical purpose, we often need to bind its behaviors, but it should be achieved *via* the control of the system’s experience, rather than by designing the system in a special way, as also described in the study by Bieger and Thórisson (2016).

### 3.3 Mental Operations

An operation may be completely executed by the actuator of the host system (e.g., a NARS-controlled robot raises a hand or moves forward) or partly by another coupled system or device (e.g., a NARS-controlled robot pushes a button or issues a command to another system). NARS has an interface for such “external” operations to be registered. Consequently, all kinds of operations to be used in a “plug-and-play” manner, i.e., to be connected to the system at run time by a user or the system itself. A learning phase is usually needed for an operation to be used properly and effectively, as NARS will gradually learn its preconditions and consequences.

In principle, operations are not necessarily demanded in every NARS implementation, except a special type of “mental” operations that operate on the system’s own “mind.” There are several groups of mental operations in the current design, including

- **Task generation:** An inference task in NARS can either be input or derived recursively from an input task. The derivation process does not change the type of the task (judgment, question, or goal). However, in certain situations, a task needs to be generated from another one of a different type. For example, a new judgment (“It is cold.”) may trigger a new goal (“Close the window!”). This relation is represented as an implication statement where the consequent is not a statement, but an operation call, similar to a production rule (Luger, 2008).
- **Evidence disqualification:** By default, the amount of evidence for every belief accumulates over time. Therefore, although the frequency value of the belief may either increase or decrease (depending on whether the new evidence is positive or negative), its confidence value increases monotonically. This treatment is supplemented by a mental operation that allows the system to doubt a belief of itself by decreasing its confidence value to a certain extent.
- **Concept activation:** The resource allocation mechanism of NARS already implements a process similar to activation spreading in neural networks (Russell and Norvig, 2010). When a new task is added into a concept, the priority of the concept is increased temporarily, and inference in the concept may cause derived tasks to be sent to its neighbors, so their priority levels will be increased, too. As a supplement, a mental operation allows the system to pay attention to a concept without new tasks added, so as to allow the system to deliberately consider a concept.

In general, mental operations supplement and influence the automatic control mechanism, and let certain actions be taken as the consequence of inference. Mental operations contribute to the system’s self-concept by telling the system what is going on in its mind and allow the system to control its own thinking process to a certain extent. For instance, the system can explicitly plan its processing of a certain type of task. After the design and implementation phases, the system needs to learn how to properly use its mental operations, just like it needs to learn about the other (external) operations.

### 3.4 Internal Experience

In NARS, “experience” refers to the system’s input streams. In the simplest implementation of NARS, the system has only one input channel, where the experience from the channel is a stream of the form *S*_1_, *T*_1_, *S*_2_, *T*_2_, …, *S_n_, T_n_*, where each *S_i_* is a Narsese sentence, with *T_i_* to be the time interval between it and the next sentence. A buffer of a constant size *n* holds the most recent experience.

In more complicated implementations, there are also “sensory” channels, each accepting a stream of Narsese terms from a sensory organ. Here, a sensor can recognize a certain type of signal, either from the outside of the system (such as visual or audio signals) or from the inside of the system, either from its body (somatosensory) or from its mind (mental). An internal channel provides a certain type of “internal experience.” Somatosensory input will be especially important for a robotic system, as it needs to be aware of its energy level, network connection status, damages in parts, etc.

A mental sensation may come from the execution of a mental operation. Also, there are mental sensations appearing as the traces of the system’s inference activity. During each inference cycle, the system “senses” the concept that was selected for processing, as well as the derivation relationship between tasks. Later, this experience can be used to answer questions such as “What has been pondered?” or “Where does that conclusion come from?,” asked either by the system itself or by someone else. This information can also be used in future inference activities.

On the input buffers, the system carries out certain perceptive reasoning to form compound terms corresponding to the spatiotemporal patterns of the input. There is also a global buffer that holds a stream of Narsese sentences that integrate inputs from all the channels. In this aspect, the external and internal experiences are handled basically in the same manner.

A special type of belief formed in perception is the temporal implications between the mental events sensed within the system and the outside events observed by the system. The system will believe that it is some of its ideas that “cause” a certain action to be performed in its environment, and such beliefs will coordinate its “mind” and its “body.” This is also arguably the origin of the notion of “causation” within the system. For a detailed discussion on temporal and causal inference in NARS, see the study by Wang and Hammer (2015).

The internal experience of NARS is the major source of its self-knowledge. Under AIKR, this type of knowledge is also uncertain and incomplete and is under constant revision. Furthermore, it is subjective and from the first-person perspective. In these aspects, NARS is fundamentally different from the “logical AI” approach toward self-knowledge, where the system is assumed as “having certain kinds of facts about its own mental processes and state of mind” (McCarthy, 1995).

### 3.5 Feeling and Emotion

According to AIKR, NARS needs to deal with different tasks with limited time and other resources. To ask the designer to provide a general optimizing algorithm to manage resources for all the possible situations is obviously impossible, and this is one of the reasons why NARS needs a mechanism to learn how to manage its resources and to make quick responses in various circumstances, all by itself. In the human mind, emotion and feeling play major roles in situation appraisal and behavior control, which are also desired in computer systems (Arbib and Fellous, 2004). In NARS, we have built a preliminary mechanism to carry out similar functions.

NARS has a basic satisfaction–evaluation mechanism at the event level. Every event has a truth value and a desire value, expressing the current status and what the system wants it to be, respectively. The closeness between them is called “satisfaction,” which indicates a basic appraisal of an individual aspect of the situation. The value of “satisfaction” is in the range [0, 1], where 0 means “completely unsatisfied,” 1 for “completely satisfied,” and the other cases are in between.

Also there is system-level satisfaction, as the accumulation of recent event-level satisfactions, which represents an appraisal of the overall situation. Technically, this value is evaluated in every working cycle by adjusting the overall satisfaction value using the satisfaction value of the event just processed. This system-level satisfaction indicates the system’s extent of “happiness” or “pleasure,” and it plays multiple roles within the system, such as influencing the resource allocation.

To make the system aware of the values of these satisfaction indicators, some “feeling” operators are implemented, which reflect these satisfaction values into the internal experience of the system, so as to involve them explicitly into the inference processes. This happens by the usage of reserved terms and statements, which form the category of “emotional concepts” within the memory of the system. These emotional concepts provide a perception of emotions within NARS to the system itself, just like how the perceptive concepts summarize the system’s experience when interacting with the outside world.

These emotional concepts interact with other concepts as generic (unemotional) concepts would, leading to the generation of compounds by the inference process, be represented by concepts that combine the emotional aspect with other aspects of the situation. Being unsatisfied about an event may be caused by other systems or the system itself, may be about the past or the future, may be controllable or inevitable, etc., and all these differences will lead to different categorization about the situation. For example, simply speaking, *regret* is the combination of negative emotion (unsatisfied situation) with other concepts like “things happened in the past” and “things caused by my own behaviors.” You will not feel *regret* about bad things that might happen in the future or caused by the behaviors of someone else.

In addition, desire value is extended to non-event concepts according to their correlation with overall satisfaction. For example, an object will be liked by the system if the appearing of this object consistently concurs with high satisfaction level, and the contrary ones will be “disliked” by the system. Of course, there are many other things for which the system has little emotion. These different attitudes mainly come from the system’s experience and will influence the system’s treatment to the concepts.

In summary, in NARS, emotional information appears in two distinct forms:

- At the “subconscious level,” it appears as desire values and satisfaction values. They are outside of the experience of the system, since these values do not form statements the system could reason about.
- At the “conscious level,” it appears as events expressed using emotional concepts. They are inside of the experience of the system, since they are represented as statements that are considered in the inference process of the system.

Emotional information in both forms contributes to the system’s internal processes, as well as to the system’s external behaviors.

The emotional concepts in experience are processed as other concepts in inference. Consequently, they categorize the objects and situations according to the system’s appraisal and allow the system to behave accordingly. For instance, the system may develop behavior patterns for “danger,” even though each concrete danger has very different sources and causes.

The “emotion-specific” treatments also happen at the subconscious level, where the emotional information is used in various processes.

- The desire values of concepts are taken into account in attention allocation, where the concepts associated with strong feeling (extreme desire values) get more resources than those with weak feeling (neutral desire values). These desire values not only help the system to judge how long data items should be stored in memory but also how much priority they should be given when under consideration.
- After an inference step, if a goal is relatively satisfied, its priority is decreased accordingly and the belief used in the step gets a higher priority because of its usefulness. This way, already satisfied goals get less attention by the system, while relevant knowledge that satisfied these goals tends to be kept in memory longer, with the related concepts “liked” by the system.
- In decisions made, the threshold on confidence is lower in high emotional situations to allow quick responses. This is especially desired in situations where there is no lot of time available to react.
- The overall satisfaction is used as a feedback to adjust the priority values of data items (concepts, tasks, beliefs), so that the ones associated with positive feeling are rewarded, and the ones associated with negative feeling punished. In this way, the system shows a “pleasure-seeking” tendency, and its extent can be adjusted by a system parameter. This pleasure-seeking tendency can be considered as a motivation that is not directly based on any task, but as a “meta-task.”
- When the system is relatively satisfied, it is more likely to create new goals, while when the system is unhappy about the current situation, it is more likely to focus on the existing goals that have not been achieved.

Overall, the system’s feelings and emotions consist of a major part of its internal experience and contribute to its self-control. Emotion also plays roles in communication and socialization, but they, as well as topics like the self-control of emotion, are beyond the scope of this article.

### 3.6 Examples

Here, we illustrate a few examples using the Open-NARS^5^ implementation of NARS. To simplify the description, the examples are slightly edited to remove the attributes not discussed in this article (such as the tense of the sentences), and before each Narsese sentence, the type of the sentence and a rough English translation are added. The ASCII symbols in the actual input/output are not the same as the logical symbols in the publications (including the above sections), but since their correspondence is hinted by their similarity and suggested by the English translation, the format will not be explained in detail, except the following:

- Judgments, questions, and goals in Narsese end with “.”, “?”, and “!”, respectively.
- Prefix “^” indicates an operator, prefix “#” indicates an anonymous term, and prefix “$” indicates a variable term that can be substituted by another term.
- When the truth value of an input judgment or the desire value of an input goal is unspecified, the default ⟨1, 0.9⟩ is used.

The first example demonstrates learning from observing the actions of another agent. Let’s assume that Michael sells a car and that it is observed that he is rich after that. Later, when the system gets the goal “to be rich,” it will want to sell a car, too, as it guesses that whatever worked for Michael will also work for itself.

Input: "Michael sells a car."
<(*,{Michael},car) --> ^sell>.
Input: "Michael gets rich."
<{Michael} --> [rich]>.
Derived: "After someone sells a car, one gets rich."
<(&/,<(*,$1,car) --> ^sell>) =/> <$1 --> [rich]>>. %1.00;0.31%
Input: "I want to be rich!"
<{SELF} --> [rich]>!
Derived: "I want to sell a car!"
<(*,{SELF},car) --> ^sell>! %1.00;0.28%

This example shows that the system uses a temporal relation as evidence for a causal relation, which of course often leads to mistakes. In NARS, such mistakes are corrected by further negative evidences, that is, when the system learns other car-selling events that do not bring richness to the seller. This is also how the system resolves competing explanations and predictions, that is, by accumulating evidence on the competing hypotheses and choosing the best supported one.

The next example illustrates how the system summarizes its experience in relation to itself. In particular, it shows that picking up trash together with the knowledge that itself is a robot leads to the formation of a compound concept that contributes to the meaning of itself as a “robot that picks up trash”:

Input: "I am a robot."
<{SELF} --> robot>.
Input: "I pick up trash."
<(*,{SELF},trash) --> ^pick>.
Derived: "I am somebody who picks up trash."
<{SELF} --> (/,^pick,_,trash)>.
Input: "What two things characterize you?"
<{SELF} --> (&,?1,?2)>?
Answer: "That I am a robot who picks up trash."
<{SELF} --> (&,(/,^pick,_,trash),robot)>. %1.00;0.81%

The intermediate result that transformed the second input statement into an *inheritance* statement about itself was crucial here. The same happens with mental operations. The case where the system wonders about whether cats are animals illustrates that:

Input: "I wonder whether cats are animals."
<(*,{SELF},<cat --> animal>) --> ^wonder>.
Input: "What am I?"
<{SELF} --> ?1>?
Answer: "I am somebody who wonders whether cats are animals.”
<{SELF} --> (/,^wonder,_,<cat --> animal>)>. %1.00;0.90%

Such a wondering event is part of the internal experience of the system and is generated by a question:

<cat --> animal>?

For this to happen, the question task needs to exceed a certain priority value, meaning the system has to consider it as sufficiently important to the current situation.

The next examples show other motivational and emotional aspects of the system, such as the usage of a “*feel*” operator. Besides that, it shows the system’s capability to consider the related event in question answering:

Input: "I don’t want to get hurt."
(--,<{SELF} --> [hurt]>)!
Input: "When running away from a close wolf, I won’t get hurt."
<(&/,<(*,{SELF}, wolf) --> close_to>,
<(*,{SELF}) --> ^run>) =/> (--,<{SELF} --> [hurt]>)>.
Input: "I am close to wolf_1 now."
<(*,{SELF}, {wolf_1}) --> close_to>.
Input: "Wolf_1 is a wolf"
<{wolf1} --> wolf>.
Execution: "I run away."
<(*,{SELF}) --> ^run>!
Input: "I did not get hurt."
(--,<{SELF} --> [hurt]>).

The system deriving that running away from the wolf is satisfying:

"Feel the amount of satisfaction!"
(^feelSatisfied,{SELF})!
Feedback: "I am relatively satisfied."
<{SELF} --> [satisfied]>. %0.65;0.90%
Input: "How can I be satisfied?"
<?how =/> <{SELF} --> [satisfied]>>?
Answer: "Running away when a wolf is close makes me satisfied."
<(&/,<wolf --> (/,close_to,{SELF},_)>,<(*,{SELF}) --> ^run>) =/> <{SELF} --> [satisfied]>>. %0.59;0.40%

Such satisfaction-related events can lead to emotion-based decisions, and, as the example shows, compound term can be composed by combining these events with other knowledge in the system.

In animals, there is usually an innate link between getting hurt by another animal and experiencing fear by future appearances of this kind of animal. Also, the response to fear, namely to run away, is usually an innate reaction and at the same time a successful strategy to survive. The following example demonstrates this case:

Innate belief: "If you are close to something that frightens you, run away"
<(&/,<(*,{SELF}, #1) --> close_to>,<(*,#1,{SELF}) --> frightens>) =/> <(*,{SELF},<(*,{SELF}) --> ^run>) --> ^want>>.
Innate belief: "If something hurts you, it frightens you."
<<(*,$1,{SELF}) --> hurt> =/> <(*,$1,{SELF}) --> frightens>>.
Innate belief: "If something frightens you, you feel fear.
<<(*,#1,{SELF}) --> frightens> =|> <(*,{SELF},fear) --> feel>>.
Input: "You are getting hurt by a wolf."
<(*,wolf,{SELF}) --> hurt>.

From here, it is expected that the system learned to be fearful of wolves and that it runs away whenever it encounters one.

Input: "You are close to a wolf."
<(*,{SELF}, wolf) --> close_to>.
Input: "How do you feel?"
<(*,{SELF},?what) --> feel>?
Answer: "I feel fear."
<(*,{SELF},fear) --> feel>. %1.00;0.29%
Execution: "I run away."
<{SELF} --> ^run>!

Given this encoding, the system can also be asked what frightens it:

Input: "What frightens you?"
<(*,?1,{SELF}) --> frightens>?
Answer: "The wolf frightens me."
<(*,wolf,{SELF}) --> frightens>. %1.00;0.43%

## 4 Comparisons and Discussions

In this section, the design decisions in NARS that are directly related to “self” are explained and compared with the alternatives.

### 4.1 The Need for a Self

Are self-awareness and self-control really required in an intelligent system? Why are such functions absent in most of the AI systems developed so far?

Like many controversies in AI, the different opinions on this matter can be traced back to the different understandings of “AI” (Wang, 2008). As the mainstream AI aims at the solving of specific problems, the systems are usually equipped with problem-specific algorithms, which embed knowledge about the problem domain, but not about the system itself, as the properties of the problem solver are usually irrelevant to the problem-solving process.

Even in learning systems that do not demand algorithms to be manually coded, they are still approximated by generalizing training data (Flach, 2012). In general, such systems have little need to add itself into the picture, as the solutions should only depend on the data to be learned, not the learner. Even metacognition can be carried out without an explicit “self”-concept involved (Cox, 2005)—when all the decisions are made by the system, it is unnecessary to explicitly state that.

In AGI systems, the situation is different. Here, we have projects aimed at simulating the human mind according to psychological theories, such as LIDA (Franklin, 2007) and MicroPsi (Bach, 2009), which surely need to simulate the self-related cognitive functions, simply because the well-known roles they play in human cognition (Blackmore, 2004).

In the function-oriented AGI projects, self-awareness and self-control are introduced to meet the requirements for the system, rather than sorely to be human-like. For instances, GLAIR is able to “represent and reason about beliefs about itself” (Shapiro and Bona, 2010). Sigma has the function of “architectural self-monitoring” (Rosenbloom et al., 2016). When facing varying problems, an AGI has to know itself and be able to adjust itself, so as to meet the changing situations. Since the existing AGI systems have very different overall designs, the exact form of the self-related functions differ greatly, and it is hard to compare and judge them in details without taking the whole system into consideration.

In general, NARS is more similar to GLAIR and Sigma than to LIDA and MicroPsi, as it is designed to realize a certain understanding of intelligence, which is generalized away from its realization in human beings. For NARS, the need for self-awareness and self-control follows from its working definition of intelligence, that is, adaptation under AIKR (Wang, 2008). To adapt to the environment and to carry out its tasks, the system needs to know what it can do and how it is related to the objects and other systems in the environment, and an explicitly expressed *SELF*-concept organizes all the related tasks and beliefs together, so as to facilitate reasoning and decision-making.

It may be argued that there is already a “self” in many AI systems, as knowledge in the system is often conceptually “about itself,” “by itself,” or “for itself.” Why bother to explicitly spell that out and to separate it from other knowledge?

It is indeed the case that many AI systems have self-knowledge without explicitly talking about itself, but taking it as the default. For example, many works under the name of “metacognition” (Cox, 2005) have knowledge about various algorithms within the system itself and use this knowledge to select a proper one for the current problem. Although this process is self-reflective by nature, the systems typically does not have an explicitly represented “self.” Instead, the processes are separated into “object-level” and “meta-level,” where the latter monitor and control the former (Cox, 2005; Marshall, 2006).

Although an “implicit self” is enough for many problems, an explicitly represented *self*-concept provides many advantages desired in general-purpose AI that must adapt to various situations. This idea is not really new, as it can be at least traced back to McCarthy (1995), who promoted the idea of “making robots conscious of their mental states.” In NARS, the *SELF*-concept provides a flexible unit for the representation and processing of self-knowledge coming from various sources and in different forms, although it does not cover all the self-related functions. As a reasoning system, this design allows NARS to uniformly represent and process knowledge about the system itself and about the other systems. As shown by the previous examples, imitation can be directly carried out as analogical reasoning, by substituting another system by *SELF*.

### 4.2 Self-Awareness

The self-knowledge of NARS shares many features as the system’s knowledge about the outside environment.

All types of knowledge in NARS are organized into concepts. According to the semantics of NARS, the meaning of a concept (or a term naming a concept) is normally determined by its relation with other concepts (or terms). While for most concepts such relations are all acquired from the system’s experience, the system is not necessarily born with a blank memory. Each built-in operation contributes meaning to the concept of *SELF*, by relating the system as a whole to the events it can perceive and/or realize. Starting from these operations, the *SELF*-concept will eventually involve beliefs about

- what the system can sense and do, not only using the built-in operations, but also the compound operations recursively composed from them, as well as the preconditions and consequences of these operations;
- what the system desires and actively pursues, that is, its motivational and emotional structure;
- how the system is related to the objects and events in the environment, in terms of their significance and affordance to the system;
- how the system is related to the other systems, that is, the “social roles” played by the system, as well as the conversions in communication and interactions.

All these aspects will make the system’s self-concept richer and richer, even to the level of complexity that we can meaningfully talk about its “personality,” that is, what makes this system different from the others, due to its unique nature and nurture. It is possible to measure the complexity of a concept in terms of its conceptual relations whose truth value is stable (high *confidence*) and unambiguous (extreme *frequency*), although such a measurement does not mean much, as the intuitive richness of a concept also depends on many other factors, such as the quality and diversity of the concepts it relates to, and so on.

This treatment is fundamentally different from identifying “self” with a physical body or a constant mechanism within the system. The spatial scope of self is mainly determined by the range of the system’s sensors and effectors, which can distribute in distinct locations.

According to our approach, “self” is not left completely to a mysterious “emergent process,” neither. In NARS, the concept *SELF* starts with a built-in core, then evolves according to the system’s experience. In the process, the self-concept organizes the relevant beliefs and tasks together to facilitate self-awareness and self-control. This is consistent with Piaget’s theory that a child learns about self and environment by coordinating sensing (such as vision and hearing) with actions (such as grasping, sucking, and stepping) and gradually progresses from reflexive, instinctual action at birth to symbolic mental operations (Piaget, 1963).

NARS treats *SELF* like other concepts in the system, except that it is a “reserved word,” which has innate associations with the built-in operations, including the mental operations. NARS also treats internal and external experience uniformly, so self-awareness and self-control are nothing magical or mysterious, but are similar to how the system perceives and acts upon the external environment.

An important type of self-knowledge is provided by the emotion and feeling mechanism of NARS. As mentioned previously and described in detail in the study by Wang et al. (2016), such a mechanism is introduced into NARS, not for giving the system a “human face,” but for appraising the current situation and dealing with it efficiently.

McCarthy (1995) concluded that “Human-like emotional structures are possible but unnecessary for useful intelligent behavior.” We agree that “being emotional” often leads to bad judgments and undesired consequences, but still consider emotion a necessary component of advanced intelligence. Of course, the emotions in NARS are not “human-like” in details, but play similar roles as in human cognition, that is, situation appraisal and behavior control.

Due to AIKR, NARS is not aware of all of its internal structures and processes, but only the most prominent parts, “the tip of an iceberg.” Most activities within the system are beyond the scope of self-awareness, so cannot be deliberately considered. The picture is like what Freud (1965) drew about human thinking, although in NARS the unconscious processes follow the same logic as the conscious processes, except unnoticed by the system’s limited attention.

In general, NARS treats its “external experience” and “internal experience” in the same way, and the knowledge about the system itself has the same nature as other knowledge in NARS. Under AIKR, self-knowledge is incomplete, uncertain, and often inconsistent, which is the contrary of what is assumed by the “logical AI” school (McCarthy, 1995). The system can only be aware of the knowledge reported by certain mental operations and those in the input buffers, and even this knowledge does not necessarily get enough attention to reveal its implications.

### 4.3 Self-Control

Although the system only has limited self-knowledge, it nevertheless make self-control possible.

First, it is necessary to clarify what “self-control” means in this context. As almost all control activities are carried out by the system and the results are often within the system, to consider all of them “self-control” would trivialize the notion. Instead, the label should be limited to the actions resulting from the system’s case-by-case reflective and introspective deliberation, rather than from the working routines that are in the system’s initial design, as the latter should not be considered as the decision “by the system itself,” but by the designer of the system.

A widely agreed conclusion in psychology is that a mental process can be either *automatic* (implicit, unconscious) or *controlled* (explicit, conscious), with respect to the system itself. The former includes innate or acquired stimulus–response associations, while the latter includes processes under *cognitive control*, such as “response inhibition, attentional bias, performance monitoring, conflict monitoring, response priming, task setting, task switching, and the setting of subsystem parameters, as well as working memory control functions such as monitoring, maintenance, updating, and gating” (Cooper, 2010). Various “dual-process” models have been proposed in psychology to cover both mechanisms, such as the study by Kahneman (2011). Such models are also needed in AI, even though the purpose here is not to simulate the human mind in all details, but to benefits from the advantages of both. In general, controlled processes are more flexible and adaptive, while automatic processes are more efficient and reliable. Such a model often uses meta-level processes to regulate object-level processes (Cox, 2005; Marshall, 2006; Shapiro and Bona, 2010; Rosenbloom et al., 2016), and such works are also covered in the study of machine consciousness (Chella et al., 2008; Baars and Franklin, 2009).

Even though this “object-level vs. meta-level” distinction exists in many systems, the exact form of the boundary between the two levels differs greatly, partly due to the architectures involved. A process should not be considered “meta” merely because it gets information from another process and also influences the latter, since the relation can be symmetric between the two, while normally the object-level processes have no access to the meta-level processes.

As a reasoning system, in NARS, “control” means to select the premise(s) and the rule(s) for each inference step, so as to link the individual inference steps into task-processing processes. The primary control mechanism of NARS is coded in a programming language and is independent of the system’s experience. It is automatic and unconscious, in the sense that the system does not “think” about what to do in each step, but is context driven and data driven, while the data involved come from selections biased by dynamic priority distributions. On top of this, there are mental operations that are expressed in Narsese and invoked by the system’s decisions, as a result of “conscious” inference activities. This meta-level deliberative control does not change the underlying automatic routines, but supplement and adjust them. This design is different from the metacognition implemented in the other systems (Cox, 2005) in that the operations in NARS are light weight and can be accomplished within a constant time, rather than decision-making procedures that compare the possible actions in detail with a high computational cost. In this aspect, they are similar to the “mental acts” in GLAIR (Shapiro and Bona, 2010).

Like the situation of self-awareness, in NARS, self-control is far from “complete” in any sense, because of AIKR. The system can only make limited adjustments in its control mechanism, so cannot “completely reprogram itself” and nor can it guarantee the absolute correctness of its self-control decisions, as they are based on the experience of the system, while the future can be different.

### 4.4 Self-Organization

There are processes in NARS where the *SELF*-concept and mental operations are not directly involved although the related issues are usually involved in the discussions related to “self.”

One natural expectation for AI systems is that their functions and capabilities should not be completely “handcrafted,” but self-constructive and self-organizing (Simon, 1962; Thórisson, 2012). We share this opinion, and therefore in NARS, “self-organization” and “learning from experience” refer to the same group of activities, which happens in various aspects of the system:

- **Knowledge.** According to experience-grounded semantics, the “knowledge” of NARS is not an objective description of the environment, but a summary of the system’s subjective experience. The sensory experience is restricted by the system’s sensors and its social experience by its linguistic capability and communicational channels. Furthermore, the system does not merely remember whatever it has experienced, but selectively keeps them, and generates conclusions and concepts to summarize and generalize the experience, so as to deal with new situations efficiently. NARS is not a traditional “symbolic system” that merely refers to the objects and events existing outside. Instead, the concepts and statements capture the regularities and invariants in its experience, so are fundamentally from the view point of the system itself. For an object, what the system knows is not its objective characters, but is “affordance” to the system, using the vocabulary of Gibson (1986).
- **Skill.** A special type of knowledge is the *skills*, i.e., procedural knowledge guiding the usage of the system’s operations. As described previously, each operation is evoked when a certain condition is satisfied, and compound operations can be formed. Although some of such knowledge is innate, similar to the primitive reflexes of human beings, they nevertheless can be modified by the system’s experience. Among all possible compounds, which ones will be actually formed also depends on the system’s experience, like skill acquisition in humans. NARS has the ability of self-programming, in the sense that the system can organize its atomic operations into compound operations recursively and use them as a whole, so as to avoid repeated planning or searching (Wang, 2012b). In this aspect, NARS is similar to the “recursive self-improvement” model in the study by Steunebrink et al. (2016).
- **Motivation.** The motivational structure of the system is under constant adjustments and developments and is not fully specified by its designer or users. NARS is built to accept any task expressible in Narsese in any time, although the priority of each task will be adjusted by the system, and the system may even ignore some given tasks, as the consequences of conflict resolution, preemptive action, redundancy reduction, etc. From the given tasks and the system’s beliefs, derived tasks are generated recursively *via* backward inference, initially as means to achieve the given tasks, but may gradually become autonomous. As the system “grows up,” its motivational structure gradually evolves, and all the tasks in it collectively decide what the system desires at the moment. Therefore, the goals and drives of the system are determined by the system’s design, the given tasks, and the experience of the system, but not by any of these factors alone (Wang, 2012a).

In summary, there is a relatively clear distinction between *object-level* and *meta-level* in NARS, where the former is specified in Narsese and formed *via* self-organization, while the latter is specified in the programming language (such as Java) and mostly independent of the system’s experience.

Since all aspects of the object-level can be learned, everything expressible in Narsese is learnable, in the sense that it can be entered into the system, derived by the inference rules, as well as modified by new experience. Consequently, NARS is more sensitive to its experience than most AI systems developed so far, and learning happens in several different forms in various parts of the system. This treatment of learning is fundamentally different from the current machine learning paradigm (Russell and Norvig, 2010; Flach, 2012), since in NARS the learning processes do not follow algorithms and nor do they necessarily produce problem-specific mappings (Wang and Li, 2016).

This sensitivity to experience does not mean pure subjective or arbitrary behaviors. The objectivity in knowledge comes from communication and socialization. Generally speaking, the more a NARS-based system communicates with other systems and humans, the more objective it usually becomes, and the less its idiosyncratic experience matters, because its beliefs are based more on the common experience shared by the community it belongs to, although it is hard, if not impossible, to quantify this “extent of objectiveness.”

On the other hand, in NARS, the meta-level knowledge is built into the system and immune to experience-triggered modification. This level includes the grammar rules of Narsese, the inference rules of NAL, the basic routines of memory management and inference control, the set of mental operators, etc. Even taken self-awareness and self-control into consideration, this built-in core is still fixed. As stated in the study by Hofstadter (1979), “Below every tangled hierarchy lies an inviolate level.” Some approaches of recursive self-improvement suggest more radical and thorough self-modifications, but they usually ignore AIKR by assuming that the system can be sure that its self-modification can really improve its performance and that the system can afford the computational cost of complex deliberation and modifications needed for such improvements (Schmidhuber, 2007; Goertzel, 2014). We consider such assumptions unrealistic and therefore is irrelevant to the design and development of AGI systems.

### 4.5 Consciousness

Among the issues related to “self,” *consciousness* is probably the most confusing one. This topic can be addressed from many different perspectives (Blackmore, 2004), and there is still less consensus on its basic form and function. Many people consider it impossible in AI, although there have been attempts to produce consciousness in computers (Baars and Franklin, 2009) or robots (Chella et al., 2008), based on various interpretations of the notion.

Here, we focus on the so-called hard problem, that is, how physical processes in the brain give rise to subjective experience (Chalmers, 1996). Our position, briefly speaking, is that the problem is not between “physical process” and “subjective experience” but between different types of experience.

As explained previously, the experience-grounded semantics (EGS) of NARS defines truth value of statements and meaning of concepts according to the system’s experience and therefore rejects the assumption of an “objective description” of the world that is independent of any observer. Although the *world* (or call it “environment,” “universe,” etc.) exists independently of any observer, a *description* of it does not. First, a sensation is produced by a sensor; then, a perception depends on the generalization and association capability and the available concepts of the observer; finally, when the perception eventually becomes a description, the system must have paid enough attention to it, which in turn demands a relevant motivation, a proper emotional status, and so on. Therefore, there is no description that is from the viewpoint of nobody and describes the world “as it is.” The so-called objective description is nothing but the shared opinions among human beings formed from communication, socialization, education, and so on, so it is not from any single person’s viewpoint, but that of a human society. Therefore, this “objective” is actually “intersubjective” (Gillespie and Cornish, 2009). Beside the culture heritage, our descriptions of the world heavily depend on the common sensorimotor mechanism of the human species, which is not necessarily shared by all cognitive systems, like the other animals or robots, either the existing ones or the future ones.

Nagel (1974) raised the question of “What is it like to be a bat?,” which has an obvious analogy in AI, “What is it like to be a robot?” As the sensorimotor mechanisms of robot are not identical to those of the human beings, we should not expect them to form concepts whose contents are exactly the same as human concepts, although through communication with human, shared concepts with overlapping meaning are possible to various extents, depending on the design of the robot and its training and working environment. This conclusion is not limited to robots. Actually EGS can be applied to any system, as far as it has interaction without its environment. For such a system to become “grounded,” “embodied,” or “situated,” the key is not whether its input/output mechanisms are “human-like,” but whether its behaviors depend on its experience (Wang, 2009).

A direct implication of the above conclusion is that intelligent systems in the same world may form different descriptions of the world, due to their different sensorimotor organs, concept repositories, motivational orientations, etc., even when their cognitive mechanisms are basically the same. In this situation, all these descriptions are valid, even when they are incommensurable. This is not saying that any arbitrary description is valid, but that its validity can only be evaluated according to the system’s configuration and experience, rather than according to “the facts.”

The same is true within the same system. If the system applies two different sets of sensorimotor mechanisms to the same process, it may get two descriptions, which are correlated, but incommensurable, and cannot be reduced into each other. We believe that this is exactly where the “explanatory gap” comes in consciousness.

As described above, NARS has internal experience about what is going on inside the system, which directly comes from the mental operations and the related introspective functions. When the system also learns how its own design works from a third-person perspective, even when it is given a way to observe its own running process at the machine language level, it will also have two incommensurable descriptions with a gap in between. In this case, it is incorrect to consider the high-level (mental) descriptions as “raised from” the low-level (physical) descriptions, as the latter is not “more real” than the former in some sense. This position also rejects the possibility of “zombies” that behave just like us, but have no consciousness (Chalmers, 1996), because if the system does not have internal experience, it will lack certain cognitive functions and therefore will not behave just like conscious beings.

In summary, we believe that the design of NARS enables the system to have consciousness, and the related phenomena can be explained without being reduced into phenomena in neuroscience (Koch, 2004) or quantum physics (Penrose, 1994). In AGI systems, although initially the conscious functions will be relatively simple and poor, they will become more and more complicated and rich, as the research progresses. The fact that we cannot directly sense them cannot be used to deny their experience, just like one cannot deny the consciousness of another person simply because one cannot directly know what it is like to be that person.

## 5 Conclusion

Self-awareness and self-control are important cognitive functions needed by advanced AGI systems (Chella and Manzotti, 2012). For a system to solve various types of problems, especially novel ones, it needs to know about itself, as well as to adjust its own working processes, so as to efficiently produce the best answer it can find with the current evidence and resource supply.

Just as a system’s knowledge and control of its external environment are usually incomplete and fallible, so are its knowledge and control of its internal environment. An AGI system can learn how itself works using its introspective capability, especially the mental operations. It can also deliberately invoke some mental operations to realize the system’s decisions and to adjust its working procedures. These functions enable the system to better adapt to its environment and to carry out its various tasks more efficiently. Even so, it can never fully know itself nor can it have complete self-control.

Although the study of self-awareness and self-control in NARS is still at an early stage, the conceptual design described above has been implemented, and is under testing and tuning. There are many details to be refined, and many self-related issues to be further explored, like those discussed in the studies by Hofstadter (1979) and Blackmore (2004). We believe the overall design is in agreement with the scientific knowledge on these processes in the human mind and also meets the needs and restrictions in AGI systems. We also believe that almost all self-related functions observed in the human mind will be reproduced in AGI systems in principle although the details will be different. Furthermore, these functions should not be modeled one by one in isolation, but all together according to the same basic principles of intelligence.

## Author Contributions

PW proposed the overall structure and drafted Sections 1, 4, and 5. XL drafted Section 2. PH drafted Section 3. All authors revised the whole article.

## Conflict of Interest Statement

The authors declare that the research was conducted in the absence of any commercial or financial relationships that could be construed as a potential conflict of interest. The reviewer, HD, and handling editor declared their shared affiliation.

## Footnotes

^1^This treatment is similar to the set-theoretic definition of “relation” as set of tuples, where it is also possible to define what is related to a given element in the relation as a set. For detailed discussions, see the studies by Wang (2006, 2013).

^2^The definitions of disjunction and conjunction in propositional logic do not require the components to be related in content, which lead to various issues under AIKR. In NARS, such a compound is formed only when the components are related semantically, temporally, or spatially. See the study by Wang (2013) for details.

^3^Here, the direction of the arrowhead is the direction of the implication relation, while the direction of the slash is the direction of the temporal order. In principle, copulas like “/⇐” can also be defined, but they will be redundant. For more discussion on this topic, see the study by Wang (2013).

^4^The truth value function of the choice rule and the syntactic complexity of a term is defined in the study by Wang (2013).

^5^Source code, working examples, and documentations of Open-NARS can be found at http://opennars.github.io/opennars/.
